# Cortical functional connectivity and topology based on complex network graph theory analysis during acute pain stimuli

**DOI:** 10.1117/1.NPh.12.2.025010

**Published:** 2025-05-14

**Authors:** Yijing Luo, Jiaohao Du, Fanfu Fang, Ping Shi

**Affiliations:** a University of Shanghai for Science and Technology, School of Health Sciences and Engineering, Shanghai, China; b Changhai Hospital, Naval Medical University, Department of Rehabilitation Medicine, Shanghai, China

**Keywords:** acute pain, functional brain network, graph theory, small-world properties, functional connectivity

## Abstract

**Purpose:**

We aimed to investigate alterations in the topological organization of functional brain networks in acute pain.

**Methods:**

A total of 29 capsaicin group (CAP) and 19 sham controls (Sham) underwent a 10-min resting-state functional near-infrared spectroscopy scan. The CAP group applied capsaicin cream (0.1%) to the lower back, whereas the Sham group applied a hand cream without capsaicin ingredients to the same area. All subjects were healthy individuals prior to the experiment and did not report any pain or other medical history. The pain in the CAP was only caused by the topical application of capsaicin. Each subject was asked to complete a numerical rating scale. Graph theory–based analysis was used to construct functional connectivity (FC) matrices and extract the features of small-world networks of the brain in both groups. Then, FC differences in the prefrontal cortex were characterized by statistical analysis, and the altered brain features were explored.

**Results:**

Compared with Sham, CAP had impaired functions in short- and long-distance connectivity (p<0.05). In particular, there was a greatly significant difference in connectivity associated with the left dorsolateral prefrontal cortex (ldlpfc) (CAP versus Sham: 0.80±0.02 versus 0.70±0.05, p<0.0001). Global efficiency, local efficiency, and small worldness were significantly lower in the topological parameters in CAP than in Sham (CAP versus Sham: 0.172±0.018 versus 0.191±0.015, t=3.758, p=0.0005; 0.253±0.012 versus 0.283±0.012, t=8.209, p<0.0001; 0.526±0.031 versus 0.628±0.082, t=3.856, p=0.0009). At the regional level, there were deficits in nodal efficiency within the medial prefrontal cortex and ldlpfc (CAP versus Sham: 0.156±0.081 versus 0.175±0.067, t=2.305, p=0.0257; 0.169±0.089 versus 0.156±0.081, t=2.194, p=0.0033).

**Conclusions:**

Even brief episodes of acute pain can significantly reshape the brain’s network architecture and FC, revealing a complex phenomenon beyond a transient sensory experience. Disruptions in brain network topology and connectivity due to pain suggest potential avenues for targeted therapeutic interventions and a reconfiguration of brain networks that could underlie chronic pain formation.

## 1 Introduction

Pain is the body’s protective response to potential injury and involves complex physiological and neural mechanisms. The brain processes pain signals not only through nociceptive pathways but also via emotional and cognitive integration, resulting in a highly subjective and variable experience.^1^ In addition, the representation of pain in the brain tends to be complex and dynamic by nature.^2^ Notably, the prefrontal cortex (PFC) plays a crucial role in both pain processing and the regulation of higher order functions, adding another layer of complexity to our understanding of pain perception.

The use of neuroimaging techniques [e.g., functional magnetic resonance imaging (fMRI), positron-emission tomography (PET), electroencephalography (EEG), and functional near-infrared spectroscopy (fNIRS)] has become indispensable recognition due to their capabilities to explore human brain structure and function and key mechanisms involved in pain processing. fMRI has become the most widely used brain imaging technique in the field of pain research due to its whole-brain observation and good spatial resolution.^3^^–^^5^ However, the strict limitations of fMRI on head movement and the noisy environment of the closed and small area make it difficult to use in awake children, infants and young children, claustrophobic patients, and patients with some long-term bedridden conditions. Furthermore, the cost of acquiring and operating fMRI systems limits its use in large-scale studies. Previous studies have shown that fNIRS, with superior portability, enhanced temporal resolution, and greater tolerance to movement artifacts, is more suitable for investigating brain function in naturalistic settings and among special population.^6^^–^^9^ This makes fNIRS technology ideal for clinical testing. Moreover, compared with fMRI, fNIRS directly measures oxyhemoglobin (HbO) dynamics with higher temporal resolution (10 to 100 ms), rapid signal stabilization,^10^ and lower operational costs^11^ while maintaining ease of use. Several studies simultaneously using fNIRS and other technologies have demonstrated the validity and reliability of fNIRS signals, thus providing an empirical basis for its application.^12^^–^^14^

In the field of pain research, fNIRS studies have demonstrated that external pain stimuli in healthy and diseased patients evoked changes in oxygenation levels in distinctive cortical regions.^15^^–^^18^ Some studies report alterations in task-based functional activation or functional connectivity (FC) associated with pain.^19^^–^^21^ FC quantifies statistical dependencies among neural time series from distinct brain regions, reflecting synchronized activity. Because resting-state functional connectivity (rsFC) captures intrinsic brain network organization independent of tasks, making it a cornerstone of connectomics research. The rsFC is commonly used as a biomarker of neural mechanisms. For instance, in fibromyalgia patients, the rsFC between the default mode network and pain matrix brain regions was enhanced, and this alteration was correlated with clinical indicators such as the patient’s pain level and pain duration. This implies that rsFC has the potential to be used as a biomarker for assessing the severity of the condition and response to treatment in patients with chronic pain.^22^ Some acute pain studies have observed rsFC changes in healthy volunteers during experimental pain stimuli (e.g., heat and mechanical).^23^^,^^24^ During the stimuli, rsFC changed rapidly in pain- and emotion-related brain regions and recovered after the stimuli ceased, suggesting that rsFC may reflect real-time neural activity in acute pain and serve as a biomarker of its neural mechanism. Although prior work has mapped FC within pain-related cortical regions, graph theoretical analysis of fNIRS data remains scarce, particularly for acute pain’s impact on global network efficiency and local hub integrity.

Leveraging these advancements, this study aims to investigate the abnormal topological properties and FC of brain regions during acute pain states. By applying graph theoretical analysis to resting-fNIRS data, we seek to provide new imaging evidence for neurological alterations in individuals experiencing acute pain, ultimately contributing to a deeper understanding of pain mechanisms and the development of targeted therapeutic interventions.

## 2 Materials and Methods

### 2.1 Subjects

A total of 56 healthy volunteers were recruited for this study and randomly assigned to the capsaicin group (CAP) (n=35; 14 female, 21 male) or the sham controls (Sham) (n=21; 10 female, 11 male). The mean age of the subjects was 25.3±2.1 years. The larger number of subjects with CAP was chosen to exclude those who might not respond to topical application of capsaicin cream^25^ or excessive head movements during the fNIRS scan due to discomfort.^26^ All subjects were right-handed. None subjects reported any acute or chronic pain or other medical history. Exclusion criteria were drug intake during the previous week, except for vitamins. Subjects were asked to avoid caffeine prior to the experiment. Informed consent was obtained from all subjects before the study began. The study had the approval of the Shanghai Changhai Hospital Ethics Committee (ChiCTR2400087894).

### 2.2 Capsaicin

Capzasin-HP cream was purchased from America and contained capsicum oleoresin BPC 1973 at 12.5% in Unguentum M (equivalent to 0.1% capsaicin).

### 2.3 General Experimental Procedure

All study procedures were conducted in a quiet and dimly lit room. Upon arrival at the laboratory, subjects were informed of the complete experimental procedure and the precautions to be taken. Next, capsaicin or hand cream was applied to the subject’s lower back (lumbar 2 to lumbar 4 paravertebral muscles). For subjects in CAP, 1-mL Capzasin-HP cream (0.1% capsaicin) was applied to two 5×10  cm areas on the lower back. Then, the area was covered with a plastic film, which allows close contact with the skin and effectively prevented evaporation, while helping to build up body heat, resulting in a thermally abnormal pain sensation.^27^ Hand creams with the same volume but without the capsaicin property were injected into the same 5 × 10-cm skin area of the subjects. Capsaicin and hand cream were white, odorless, and of similar texture so that subjects could not distinguish between them at the start of the experiment. Before the experiment, subjects in both groups were instructed as follows, “We would apply a cream to your lower back, which may trigger varying degrees of sensation, including no sensation, a mild sensation, and even a sensation so strong that it is unbearable.”

Previous studies have shown that capsaicin cream can produce a steady and moderate-level pain 25 min after application.^27^^,^^28^ Therefore, we did fNIRS scans for 10 min, 25 min after applying the cream. The 10-min fNIRS scans were performed in the resting state, and subjects sat still with their eyes closed and tried to avoid thinking about anything else for the duration of the scan. Before and after the experiment, all subjects were asked to rate the level of pain in their lower back. The rating was made on an 11-point numerical rating scale (NRS) that extended from 0 (indicating no pain) to 10 (representing intolerable pain). During the experiment, subjects were asked to close their eyes, sit still, and try not to think about anything else.

### 2.4 fNIRS Data Acquisition and Preprocessing

**Fig. 1:**
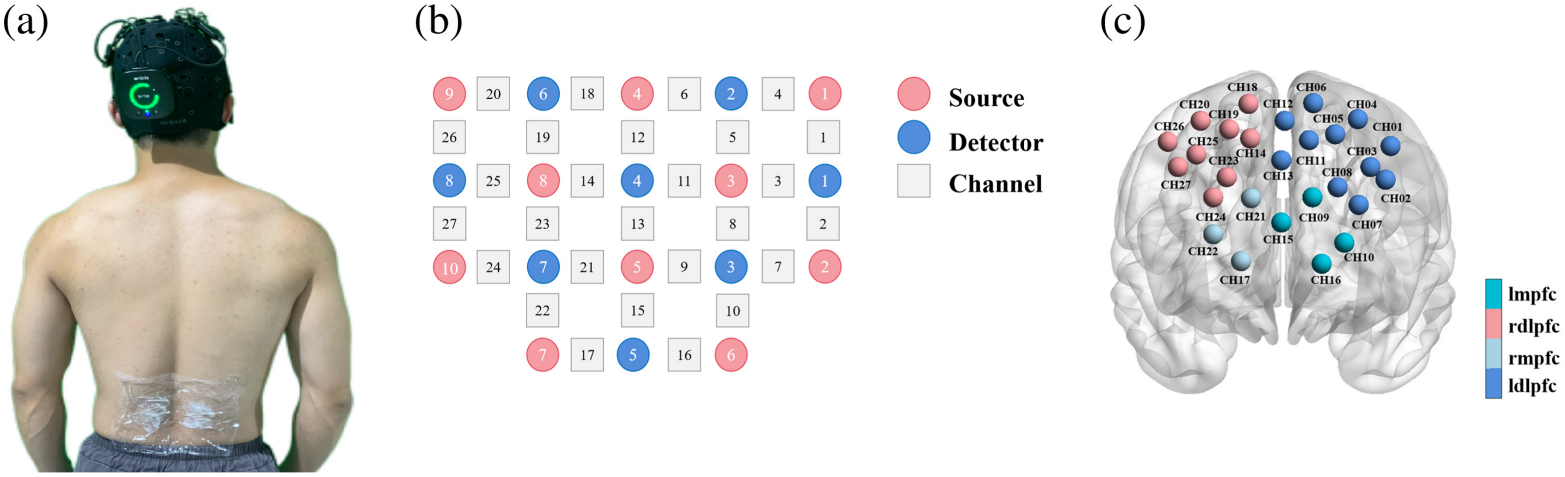
fNIRS system and channels in PFC. (a) Continuous-wave multichannel fNIRS system. (b) A 27-channel setup. (c) The oxygenated hemoglobin signals of 27 channels from three regions were detected by fNIRS.

Cerebral hemodynamic responses were recorded by means of a continuous-wave multichannel fNIRS system (Brite24, Artinis, Netherlands) [see Fig. 1(a)]. Data recording was accomplished with OxySoft software (Artinis Medical Systems, Elst, Netherlands). The signal sampling rate was 25 Hz. fNIRS system consists of 8 detectors and 10 light sources. As shown in Fig. 1(b), 8 detectors and 10 light sources form a 27-channel setup, where the maximum distance among probes was 3 cm. The fNIRS optodes were placed according to the 10 to 20 EEG system^29^ using a standardized cap (EasyCap GmbH, Herrsching, Germany). In this study, 3D spatial alignment of measurement channel positions was accomplished using a 3D digital locator (EZT-DM401) and the Broadmann atlas.^30^ The estimated positions of the channels were spatially normalized by the probabilistic alignment method in terms of the Montreal Neurological Institute (MNI)^31^ standard template. Based on this probabilistic spatial registration, the 27-channel setup covered the following cortical regions: the left medial prefrontal cortex (lmpfc) [CH9, CH10, CH15, CH16], right medial prefrontal cortex (rmpfc) [CH17, CH21, CH22], left dorsolateral prefrontal cortex (ldlpfc) [CH01, CH02, CH03, CH04, CH05, CH06, CH07, CH08, CH11, CH12, CH13], and right dorsolateral prefrontal cortex (rdlpfc) [CH14, CH18, CH19, CH20, CH23, CH24, CH25, CH26, CH27] [see Fig. 1(c)]. Table 1 shows MNI coordinates and associated brain regions of the channels together with the probability of the channels.

**Table 1:** MNI coordinates, Brodmann area, and coverage percentage of the measurement channels.

Channel	MNI coordinates			Brodmann area, coverage percentage
	x	y	z	
CH1	−42.667	24.667	50	9-Dorsolateral PFC, 0.916
CH2	−40.667	40.667	36.667	46-Dorsolateral PFC, 0.447
CH3	−34.667	41.667	41.667	9-Dorsolateral PFC, 0.720
CH4	−29.667	21.333	60.667	9-Dorsolateral PFC, 0.836
CH5	−20.667	37.333	54.667	9-Dorsolateral PFC, 0.561
CH6	−12	22.667	67	46-Dorsolateral PFC, 0.667
CH7	−30	58	26.667	46-Dorsolateral PFC, 0.862
CH8	−21.667	59	33.667	9-Dorsolateral PFC, 0439
				46-Dorsolateral PFC, 0.355
CH9	−11.667	65	29.667	10-Medial prefrontal, 0.805
CH10	−24.333	69.333	11.667	10-Medial prefrontal, 0.954
CH11	−10.333	45.667	52.333	9-Dorsolateral PFC, 0.715
CH12	−0.667	29.333	60	9-Dorsolateral PFC, 1
CH13	0.667	51.667	44.333	9-Dorsolateral PFC, 1
CH14	13	45.667	53	9-Dorsolateral PFC, 0.700
CH15	0.667	66	19.667	10-Medial prefrontal, 1
CH16	−15.333	73	3.333	10-Medial prefrontal, 0.774
CH17	16.667	73	4.333	10-Medial prefrontal, 0.858
CH18	13.667	23.333	66.667	46-Dorsolateral PFC, 0.703
CH19	21.333	36.667	56.667	9-Dorsolateral PFC, 0.692
CH20	32.667	22.667	60	9-Dorsolateral PFC, 0.762
CH21	12.667	66.667	29.333	10-Medial prefrontal, 0.875
CH22	27.667	68	15	10-Medial prefrontal, 0.989
CH23	22.333	57.333	37.667	9-Dorsolateral PFC, 0.752
CH24	27.667	60	29.667	46-Dorsolateral PFC, 0.529
CH25	34.333	39.333	46.667	9-Dorsolateral PFC, 0.968
CH26	45.667	22.333	51.667	9-Dorsolateral PFC, 0.945
CH27	41.667	38.667	41.667	9-Dorsolateral PFC, 0.613

The data preprocessing was accomplished using several functions of the Homer2 NIRS processing package^32^ in MATLAB (Mathworks, Natick, Massachusetts, United States). For each subject, the raw light intensity data series was first converted into optical density (OD). During the analysis using the enPruneChannels function with a signal-to-noise threshold of 10, channels with a very low optical intensity were discarded. Following this function, channels 1 and 4 were discarded for all subjects. Then, the motion detection algorithm hmrMotionArtifact was applied to the OD time series to identify and deal with motion-induced artifacts. This algorithm finds the data points that exceed an amplitude change threshold (AMPThresh) and a standard deviation change threshold (SDThresh) within a given time (tMotion) and then marks those points from the start of the window to tMask seconds later as motion. Both the thresholds, the window length and tMask, are set by the user. In this study, AMPThresh = 5, SDThresh = 50, tMotion = 2, and tMask = 4, which provided a compromise between the number of motion artifacts identified in noisier data series and the number identified in less noisy data series. After motion artifact identification, principal component analysis (PCA) of spatial covariance and design of spatial filters reduce artifacts common to all data channels.^33^^–^^35^ These filters were used to remove 80% of the covariance of the data using the Homer 2enPCAFilter function. hmrMotionArtifact was run again on the corrected OD time series and the trials where a motion artifact was still present were rejected. Subsequently, a band-pass filter (third-order Butterworth filter) with a cut-off frequency set at 0.01 to 0.1 Hz was applied to the data with the aim of reducing very slow drift and high-frequency noise in the data. The OD data were converted to hemoglobin concentration change values based on the modified Beer–Lambert law.^36^^,^^37^

### 2.5 Construction of Functional Brain Networks

Only HbO signals were used in the analysis because studies have shown that HbO is a more reliable indicator of cortical activation.^38^^,^^39^ FC analyses of the deoxyhemoglobin (HbR) signals were also performed, which revealed no significant differences between the two groups (CAP versus Sham: 0.37±0.03 versus 0.41±0.01, p>0.05, see the Supplementary Material). Each channel represented a node in the brain network in this study. Pearson’s correlation coefficient was calculated to construct a FC matrix to measure the linear correlation among different brain regions. Pearson’s correlation coefficient has been widely used in fNIRS studies,^11^^,^^40^^,^^41^ and its calculation is simple and easy to interpret. Although other methods (e.g., partial correlation and phase synchronization) may be advantageous in some cases, the data characteristics and sample size limitations of this study make Pearson’s correlation coefficient a more robust and appropriate choice. Subsequently, a 27×27 FC matrix was obtained for each subject. Eventually, Fisher’s r-to-z transformation was applied to convert these correlation coefficients to z-scores for improved normality. Each correlation matrix was thresholded to generate a weighted matrix with a fixed sparsity value, defined as the ratio of the total number of edges in the network to the maximum possible number of edges [Eq. (1)]. Only suprathreshold weights were retained in the resulting matrix. This sparsity-based thresholding approach ensures that the brain networks across different groups maintain an identical number of edges and consistent connection costs, thereby facilitating unbiased comparisons. Therefore, we conservatively chose a wide sparsity range from 0.05 to 0.50^42^^,^^43^ with a step size of 0.01 to fully estimate the topological properties covering the wide sparsity range. The sparsity (S) can be calculated by the following equation:

$$S=\frac{E}{E_{\max}},$$ (1)

where E is the actual number of connected edges and $E_{\max}$ is the maximum possible number of connected edges in a network with N nodes. For an undirected graph, $E_{\max}=\frac{N(N-1)}{2}$. Ultimately, we compute the area under the curve (AUC) of each topology coefficient at different sparsity thresholds as a comprehensive measure of the network topology coefficients. This approach helps to reduce the bias that may be introduced by a single threshold selection.

**Fig. 2:**
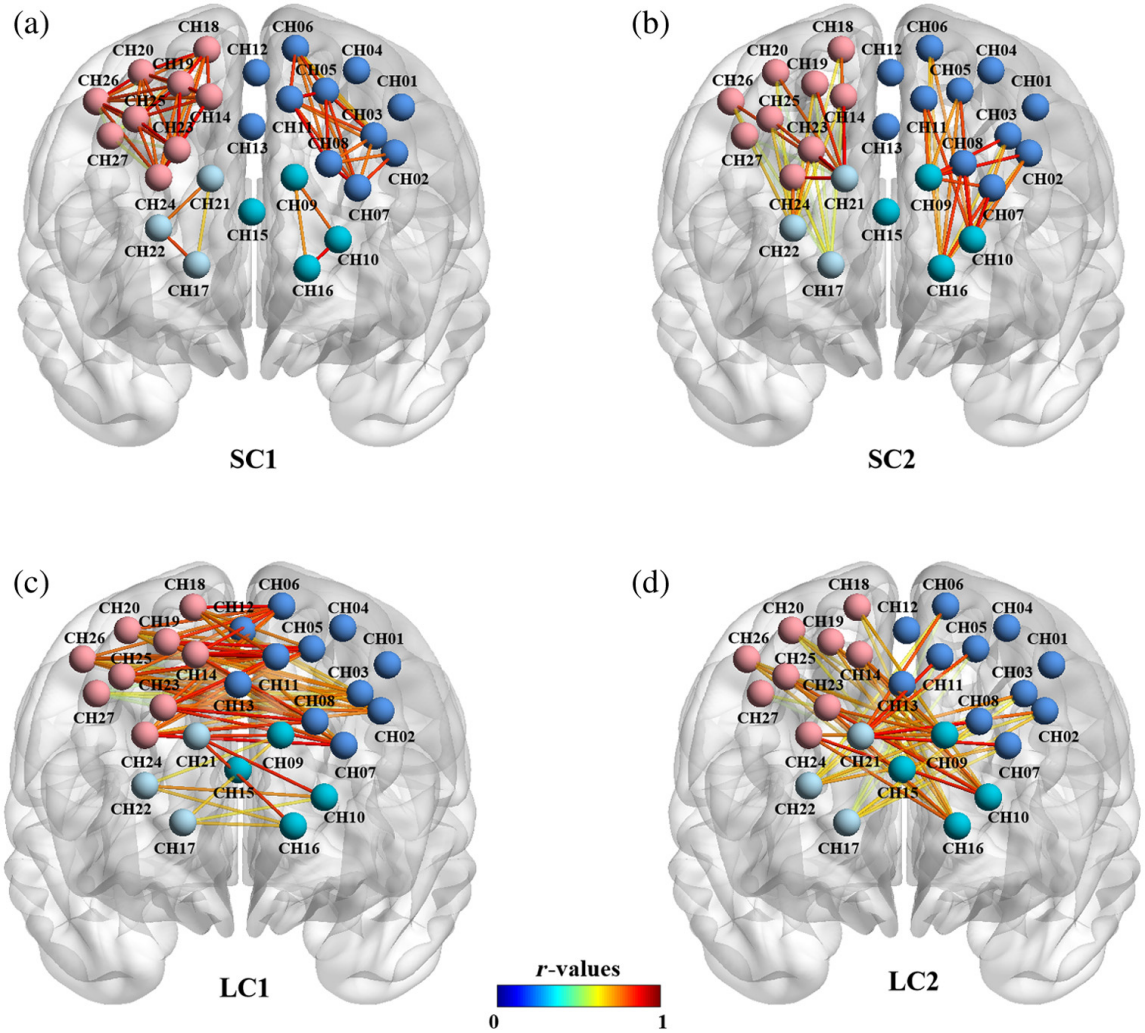
Four types of connections. (a) Short-range connectivity 1 (SC1) refers to the internal connections within the same ROI in each hemisphere. (b) Short-range connectivity 2 (SC2) refers to the connections among different ROIs within a hemisphere. (c) Long-range connectivity 1 (LC1) refers to the connections among the symmetric ROIs in different hemispheres. (d) Long-range connectivity 2 (LC2) refers to the connections among asymmetric ROIs in different hemispheres.

In region of interest (ROI) analysis, 27 measurement channels were divided into four brain regions based on their location, namely, lmpfc (CH9, CH10, CH15, CH16), rmpfc (CH17, CH21, CH22), ldlpfc (CH01, CH02, CH03, CH04, CH05, CH06, CH07, CH08, CH11, CH12, CH13), and rdlpfc (CH14, CH18, CH19, CH20, CH23, CH24, CH25, CH26, CH27). Then, the channel time series within each ROI were averaged to obtain ROI-based r-scores. The seed-based correlation was used to calculate the strength of pairwise connections at four levels in the PFC (Fig. 2): short-range connectivity 1 (SC1), denoting internal connectivity within the same ROI in each hemisphere; short-range connectivity 2 (SC2), denoting connectivity among different ROIs within a hemisphere; long-range connectivity 1 (LC1), denoting connectivity among symmetric ROIs in different hemispheres; and long-range connectivity 2 (LC2), denoting connectivity among asymmetric ROIs in different hemispheres. Pearson correlation coefficient r was converted to a normally distributed value z using the Fisher Z transformation and then used for statistical analyses. The results were visualized using BrainNet Viewer.^44^

### 2.6 Graph Theory Analyses

In this study, all network properties were computed using the analysis toolbox GRETNA. For brain networks at each sparsity threshold, we explored the topological network properties of CAP and Sham at the global and regional levels. We chose several common characteristics for the functional brain networks obtained by each participant, which cover both global and regional topological properties.

#### 2.6.1 Global network properties

The global network properties included small-world attributes clustering coefficient (Cp), characteristic path length (Lp), global efficiency (Eg), local efficiency ($E_{\mathrm{loc}}$), and small-world attributes (gamma γ, lambda λ, sigma σ).^45^^,^^46^

The Cp is an important metric in complex network analysis to measure the local clustering properties of nodes in a network. Cp reflects the closeness between a node and its neighbors

$$C_{p}=\frac{1}{N}\underset{i}{\sum }\frac{\underset{j,k}{\sum }a_{ij}a_{ik}a_{jk}}{(\underset{j}{\sum }a_{ij}-1)\underset{j}{\sum }a_{ij}},$$ (2)

where N was the number of nodes in the map. The Lp of the network quantifies the average length of the shortest path between any two nodes in a network. It is an important measure of the efficiency of information dissemination in a network

$$Lp=\frac{1}{N(N-1)}\underset{i,j\in xj\neq i}{\sum }d_{ij},$$ (3)

where $d_{ij}$ is the shortest path length between node i and node j. The Eg of network G represents the information transfer efficiency across the network, which is defined as the inverse of the harmonic mean of the shortest path length between any two nodes^47^

$$E_{g}=\frac{1}{N(N-1)}\sum_{i\neq j\in G}\frac{1}{d_{ij}},$$ (4)

where $d_{ij}$ is the shortest path length between nodes i and j. The shortest path length was the minimum number of edges included in the path that connected these two nodes. N denoted the number of nodes of the network G. Meanwhile, the $E_{\mathrm{loc}}$ of network G is defined as the average of the local efficiencies of all nodes, where the local efficiency for a given node i is the global efficiency of the subgraph composed of the nearest neighbors to node i^47^^,^^48^

$$E_{\mathrm{loc}}=\frac{1}{N}\underset{i\in G}{\sum }E_{g}(i),$$ (5)

where Eg(i) is the global efficiency of $G_{i}$, which is the subgraph of the neighbors of node i. To examine the small-world attributes of a network G that consisted of N nodes and K edges, the normalized characteristic path length ($\lambda =L/L_{\text{rand}}$) and the normalized clustering coefficient ($\gamma =C/C_{\text{rand}}$) were computed. L and C are the characteristic path length and clustering coefficient of a real network, respectively, and $L_{\text{rand}}$ and $C_{\text{rand}}$ represent the means of the corresponding parameters derived from 100 matched random networks that have the same numbers of nodes, edges, and distribution of degrees as the real brain network. Small worldness (σ) is a metric used to measure whether a network has small-world properties

$$\sigma =\frac{C/C_{\text{rand}}}{L/L_{\text{rand}}}=\frac{\gamma }{\lambda }.$$ (6)

In brief, small-world properties reflect the information exchange properties of the brain’s functional differentiation and functional integration, as well as the superb adaptive capacity of the human brain to a variety of stimuli. Typically, a small-world network should meet the following criteria: γ>1 and λ≈1 or σ=γ/λ>1.^49^

#### 2.2.2 Regional network properties

For the regional network properties, we evaluated nodal degree (ND) and nodal efficiency (NE).^48^^,^^50^^–^^53^ The ND is a simple measure of the connectivity of nodes in a network. The higher the ND, the more connections there are to that node, and the more important the node is in the network.

$$D_{i}=\sum_{j\in G}a_{ij},$$ (7)

where $a_{ij}$ represents an edge connected to node i within the network G. The NE characterizes the efficiency of information transfer between a node and its neighboring nodes,^43^ and the efficiency of node i is measured as follows:

$$NE(i)=\frac{1}{N-1}\underset{j\neq i\in G}{\sum }\frac{1}{d_{ij}},$$ (8)

where $d_{ij}$ is the shortest path length between node i and node j. Nodes with high nodal efficiency indicate that the network exhibits high tolerance to the removal of a given node, which is associated with the high clustering of that node’s neighbors.^48^

### 2.7 Statistical Analysis

Statistical and clinical data were analyzed using statistical package for the social sciences (SPSS), version 20 (IBM, Armonk, New York, United States). The normality of the data was assessed using the Shapiro–Wilk test. Independent two-sample t-tests were used to compare demographic data (except for gender ratios, analyzed by chi-square test), clinical data, and topological properties between CAP and Sham. For all statistical analyses of node topological characteristics, we implemented a false discovery rate (FDR) correction method to control for the risk of false positives due to multiple comparisons, with the significance level set at p<0.05.

## 3 Results

### 3.1 Clinical Variables

A total of 48 participants were used for formal data analyses (29 in CAP, the other 19 in Sham; 21 female, 27 male; age: 24±1.8 years). Three participants were excluded due to excessive head motion during fNIRS scanning, and another five participants had their experiments interrupted due to excruciating pain. Table 2 shows the values of CAP and Sham and mean differences with a 95% confidence interval (CI). Age, body mass index (BMI), and gender were not statistically different (p>0.05 for all comparisons made, independent samples of t-tests) between the two groups, whereas pain ratings did (6.7±1.1 versus 0.0±0.0, p<0.0001). As both groups had pain scores of 0 at the baseline level with no variability, this suggests that none of the subjects reported any pain prior to application. Thus, the changes in pain scores observed after application can be attributed to the capsaicin effects. Experimental pain induction was successful, with subjects with capsaicin cream applied to the lower back experiencing moderate to high pain levels during fNIRS scans.

**Table 2:** Demographic and pain intensity variables for CAP and Sham.

Variable	CAP (n=29)	Sham (n=19)	Statistics
Age (years)	23.7 ± 1.6	24.4 ± 2.0	t=1.364, p=0.179
BMI ($\mathrm{kg}/m^{2}$)	20.9 ± 2.7	21.5 ± 2.9	t=0.802, p=0.427
Gender (female/male)	10/19	6/14	χ2=0.108, p=0.742
Pre-application NRS	0.0 ± 0.0	0.0 ± 0.0	—
Postapplication NRS	6.7 ± 1.1	0.0 ± 0.0	t=28.67, p<0.0001

Data are expressed as mean ± standard deviation. Statistics were obtained using an independent samples t-test or Chi-square test between CAP and Sham.

### 3.2 Channel-Based Functional Connectivity

**Fig. 3:**
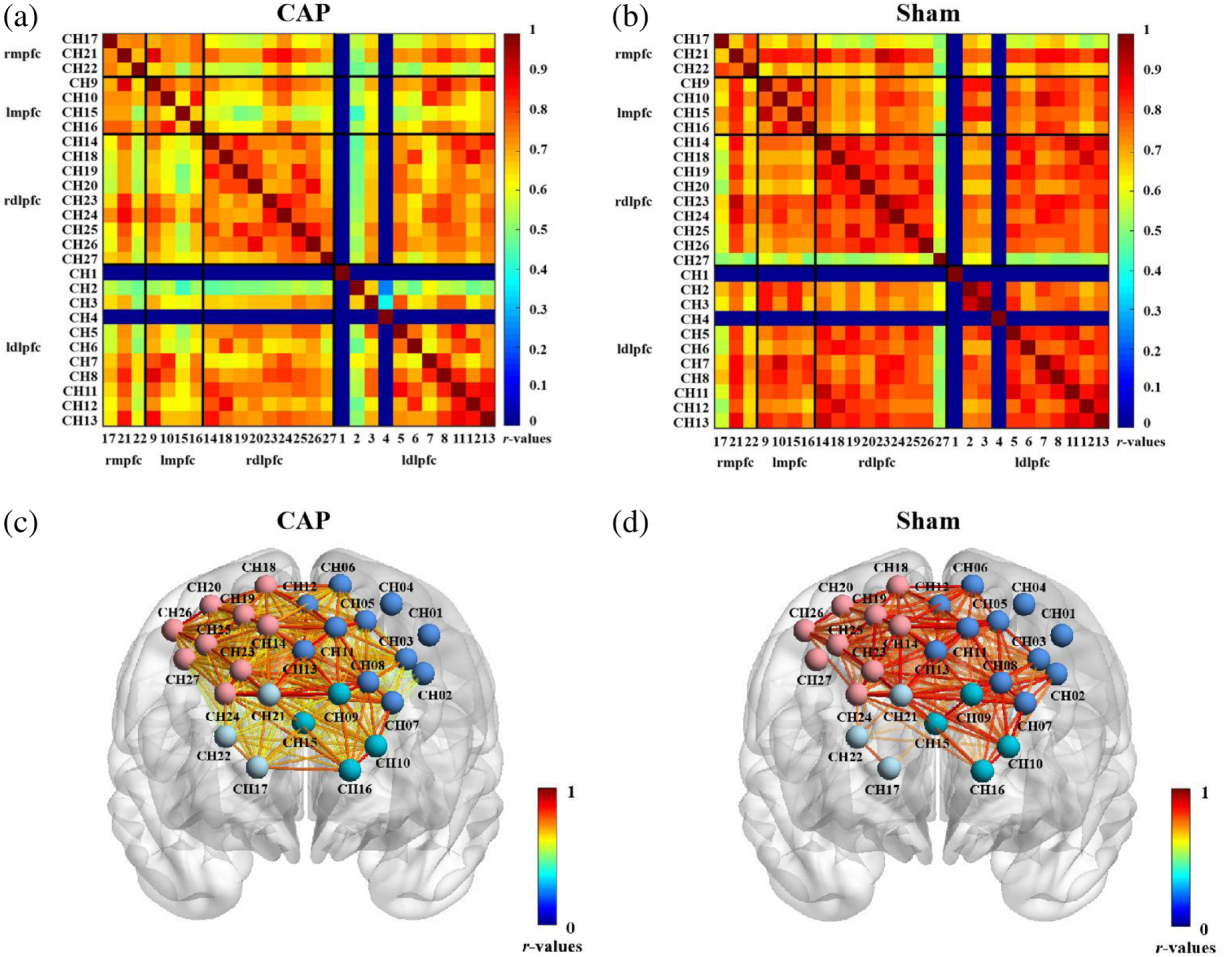
Group-average correlation matrix for CAP (a) and Sham (b). Dots represent the channels. Exclusion of channels 1 and 4 is shown in dark blue. The correlation coefficient is set to 1 (the diagonal line) for each channel. Panels (c) and (d) represent FC visualizations. Within the visualizations, nodes represent channels, and edges indicate the correlation among different channels. The color bar indicates the r-values of FC strength, with higher values indicating greater connection strength.

**Fig. 4:**
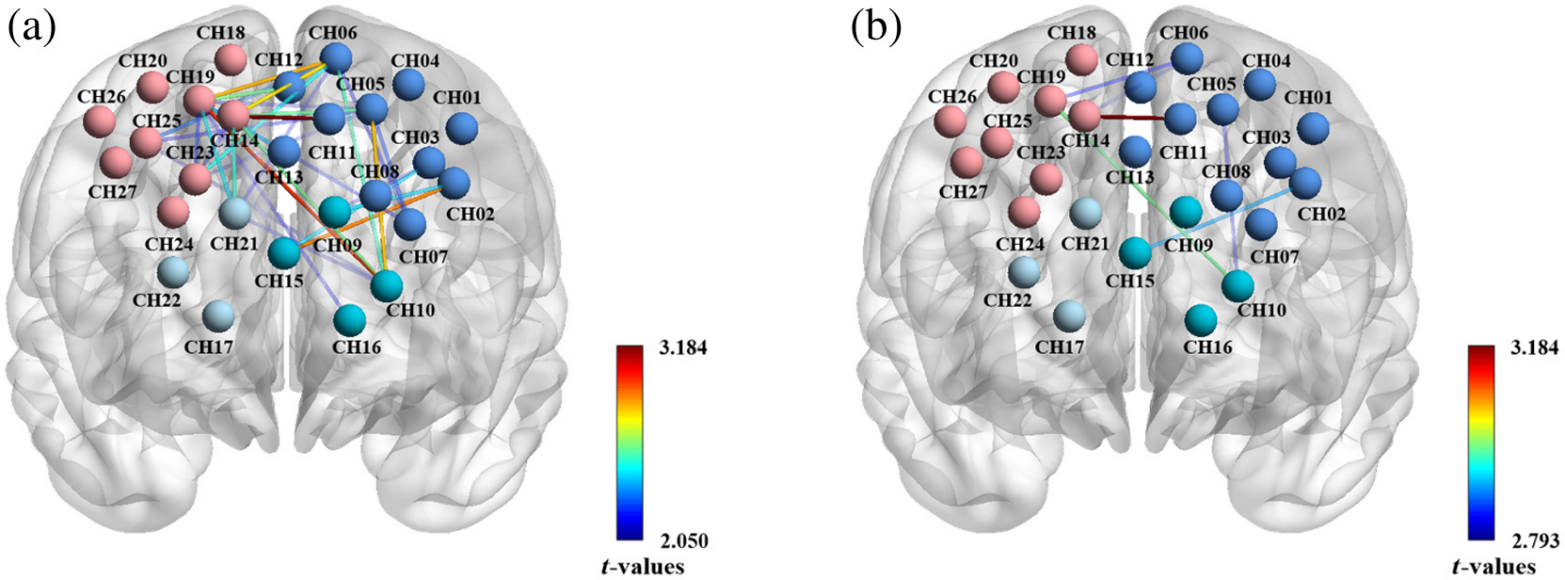
Changes in functional connections between CAP and Sham. These connecting lines represent the differences in FC strength between CAP and Sham. (a) Connections with p<0.05. (b) Connections with p<0.01. The color bar shows the t-values of the difference in FC strength. A darker red color on the line indicates a more significant difference in FC, whereas a darker blue color indicates a less significant difference.

In the group-averaged FC matrix, the spatial pattern of rsFCs was similar for the CAP and Sham (t=2.026, p=0.0479) (Fig. 3). FC was significantly attenuated in CAP compared with Sham (mean ± SD: 0.64±0.21 and 0.57±0.21, respectively). For the results of Fig. 4, there were 30 connections with a significant difference (p<0.05) and 5 connections with a highly significant difference (p<0.01). This suggests that capsaicin-induced pain may trigger a broad decline in overall brain function.

### 3.3 ROI-Based Functional Connectivity

**Fig. 5:**
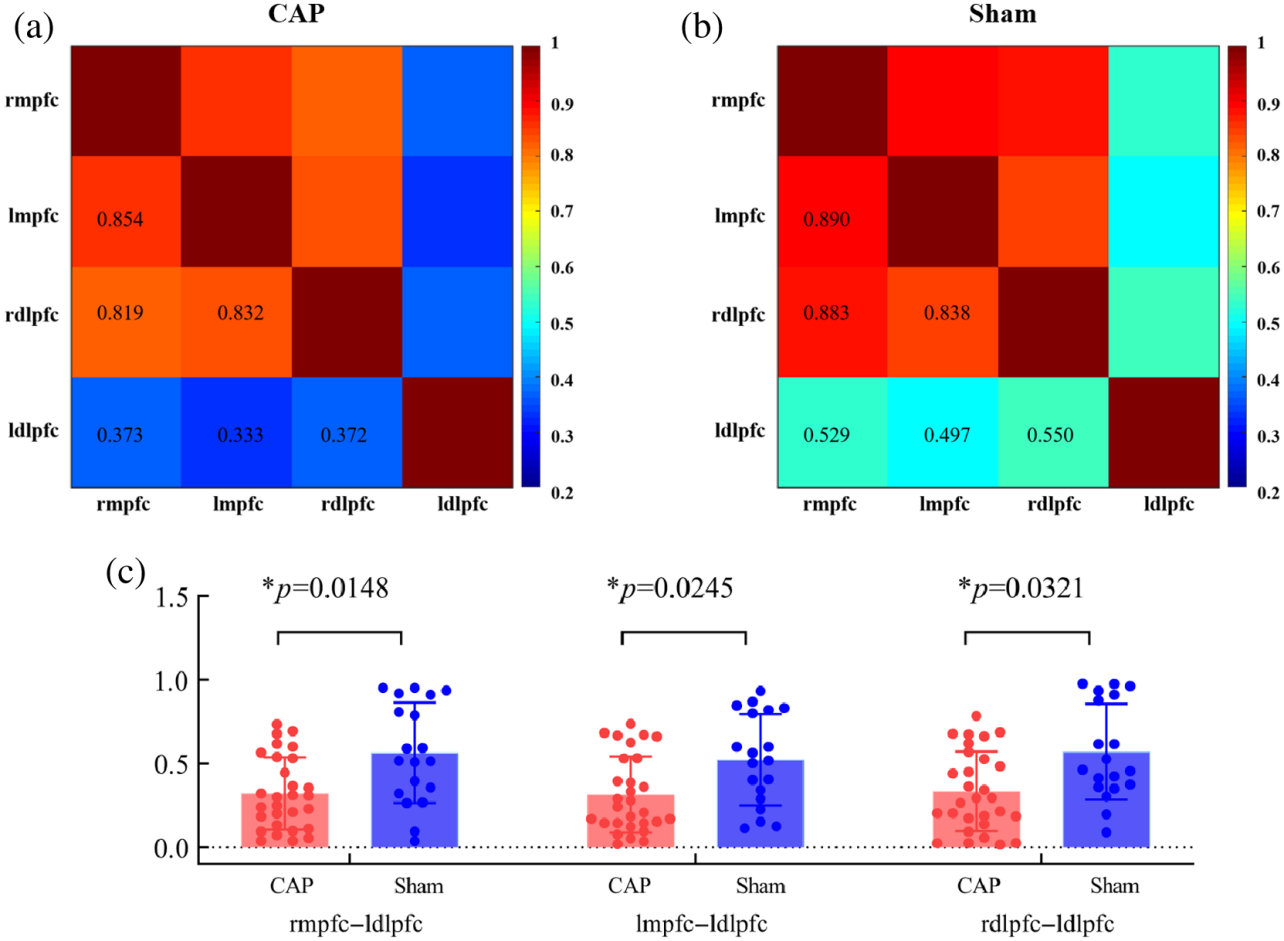
ROI-based correlation matrix for CAP (a) and Sham (b). (c) Between-group differences in ROI-based functional connections. Circles indicate the average correlation value for each ROI for each subject.

All 27 channels were divided into four ROIs to further explore the connectivity characteristics among the ROI. The time series averages of four ROIs’ internal channels were taken, and independent samples of t-tests and FDR correction were used to compare the differences between CAP and Sham. Figures 5(a) and 5(b) showed the FC matrix for ROI, with a weaker correlation in CAP. Compared with Sham, CAP had significantly lower FC intensity in rmpfc–ldlpfc (CAP versus Sham: 0.37±0.07 versus 0.57±0.04, t=2.532 p=0.0148), lmpfc–ldlpfc (CAP versus Sham: 0.31±0.09 versus 0.52±0.02, t=2.325, p=0.0245), and rdlpfc–ldlpfc (CAP versus Sham: 0.37±0.09 versus 0.57±0.01, t=2.210, p=0.0321), as shown in Fig. 5(c); there was a highly significant difference in the connectivity associated with the ldlpfc.

**Fig. 6:**
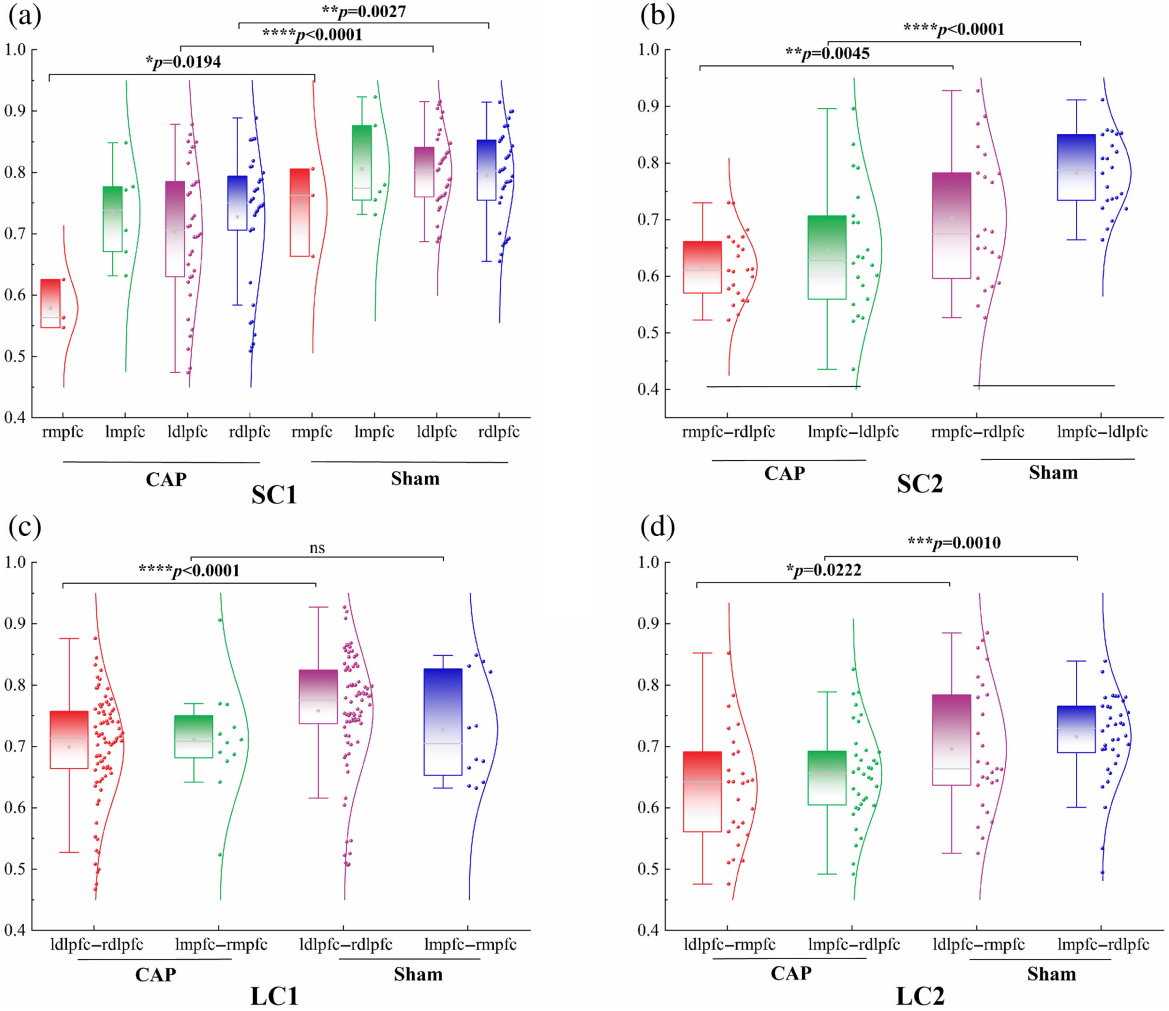
Comparison of short-and long-range connectivity between CAP and Sham. The dots indicate the correlation z-values among the ROIs. (a) Short-range connectivity 1 (SC1); (b) short-range connectivity 2 (SC2); (c) long-range connectivity 1 (LC1); (d) long-range connectivity 2 (LC2). The error bars correspond to the standard errors of the mean. *p<0.05; **p<0.01; ***p<0.001; ****p<0.0001.

Four types of FC were shown and compared among groups in Fig. 6. Both short- and long-range connectivity of CAP were weaker than Sham (p<0.05). Compared with Sham, CAP showed significantly weaker connectivity in short-range connectivity within the same ROI in each hemisphere, except for lmpfc. This was especially evident in ldlpfc (CAP versus Sham: 0.80±0.02 versus 0.70±0.05, t=4.600, p<0.0001). In short-range connectivity, CAP is significantly weaker than Sham in the left hemisphere (CAP versus Sham: 0.78±0.01 versus 0.67±0.06, t=5.515, p<0.0001). CAP showed significantly weaker connectivity than Sham in symmetric and asymmetric long-range connectivity (LC1, CAP versus Sham: 0.75±0.01 versus 0.68±0.03, p=0.0005; CAP versus Sham: 0.69±0.02 versus 0.57±0.06, LC2, p=0.0082), but not in the mpfc. In addition, there were highly significant differences in the connectivity associated with ldlpfc across these four types of FC. This is consistent with the results presented in Fig. 4.

### 3.4 Graph Theoretical Topological Analysis

**Fig. 7:**
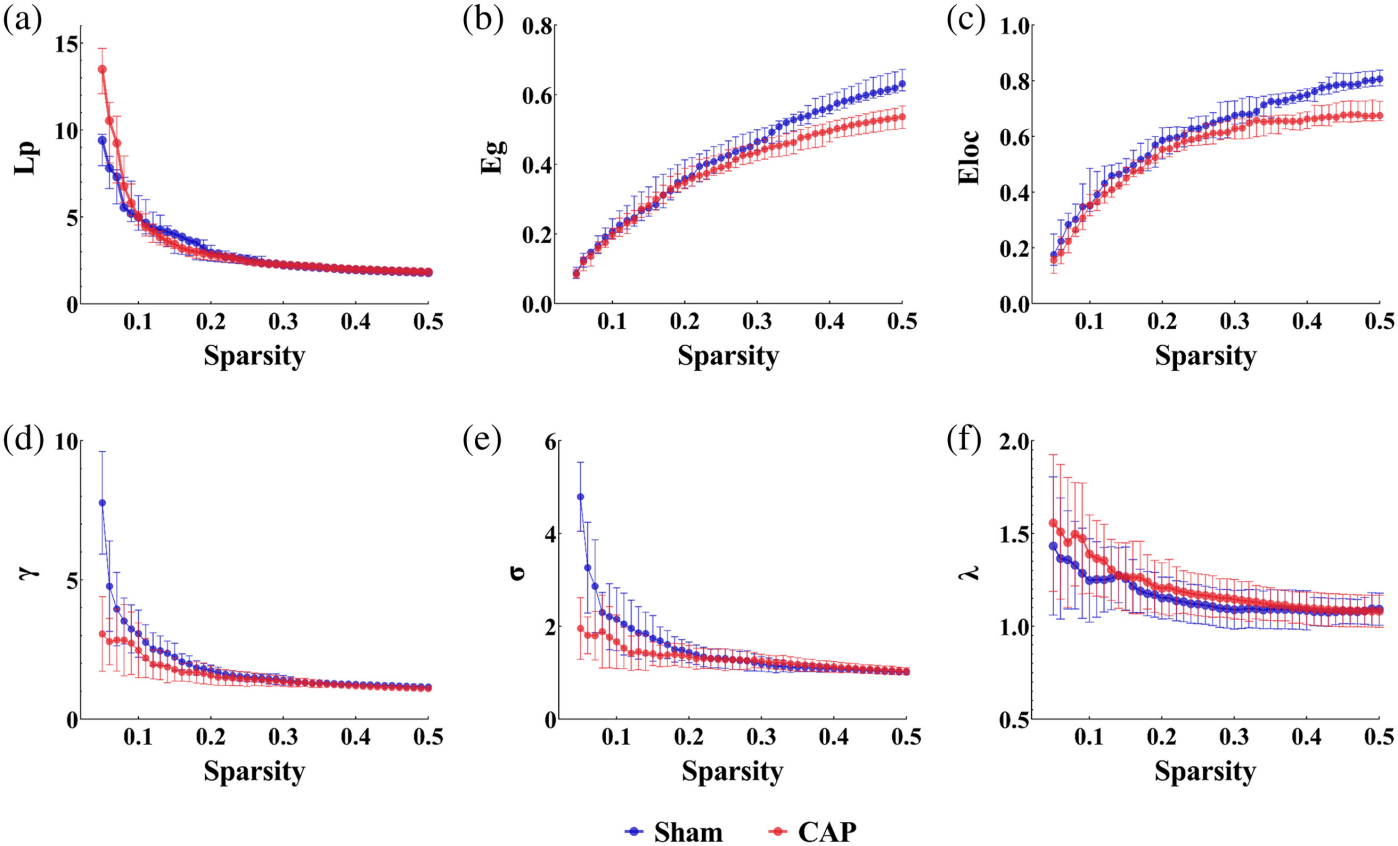
Differences in global topological properties of functional networks between Sham and CAP in the sparsity range (0.05 to 0.50). (a) Shortest path length, Lp; (b) global efficiency, Eg; (c) local efficiency, $E_{\mathrm{loc}}$; (d) gamma, γ; (e) sigma, σ; and (f) lambda, λ.

We constructed models of brain networks under the influence of capsaicin at different scales based on the FC of the brain and using threshold sparsity. The threshold sparsity ranges from 0.05 to 0.50 in 1% steps. We quantified the global network and small-world properties of the brain networks of CAP and Sham and compared the differences among them. Figure 7 demonstrates the global network features of the PFC with an increasing threshold.

Within the sparsity range of 0.05 to 0.50, we observed that changes in the levels of Lp, Eg, and $E_{\mathrm{loc}}$ were observed in all groups. Given that the data at each sparsity level (or threshold) were independent of each other, we did not find it necessary to perform analysis of variance (ANOVA) tests. We chose the independent samples of t-test and two-tailed analysis and chose a criterion of p<0.05. Specifically, within the sparsity range of 0.05 to 0.18, a significant increase in the Lp value was observed (CAP versus Sham: 1.47±0.03 versus 1.38±0.03, t=2.363, p=0.0232) [see Fig. 7(a)]. When S>0.20, the difference between the two groups was not statistically significant. On the other hand, the Eg was significantly higher in Sham than in CAP in the sparsity range of 0.22 to 0.50 (CAP versus Sham: 0.17±0.001 versus 0.19±0.004, t=3.758, p=0.0005) [see Fig. 7(b)]. At the higher sparsity range (S=0.22 to 0.50), the performance of the two groups tends to be the same, which indicates that as the sparsity increases, the network characteristics of the two groups gradually approach to 1. Meanwhile, Sham had a significantly larger $E_{\text{loc}}$ value as the sparsity increased (CAP versus Sham: 0.28±0.003 versus 0.25±0.02, t=8.209, p<0.0001) [see Fig. 7(c)]. Overall results showed that Lp was higher and Eg and $E_{\text{loc}}$ were lower in CAP compared with those of Sham.

At the defined sparsity level, all subjects showed significantly increased Cp (γ>1) and almost identical characteristic path lengths (λ≈1) for the functional brain network properties. Thus, both CAP and Sham exhibited typical small-world properties (σ>1) in all subjects in the present study. The AUCs of small-world attributes, Lp, Eg, and $E_{\mathrm{loc}}$, were significantly different between CAP and Sham (p<0.05) [Table 3 and Figs. 7(d), 7(e), and 7(f)]. Compared with Sham, we found that γ, σ, Eg, and $E_{\mathrm{loc}}$ were significantly decreased in CAP. Moreover, λ and Lp were significantly increased in CAP compared with that of Sham.

**Table 3:** Differences in the AUC of values of global network properties.

Global metrics	Sham	CAP	SMD (95%CI)	p	t
Lp	1.376 ± 0.124	1.468 ± 0.134	0.125 (–0.170, 0.013)	0.0232	2.363
Eg	0.191 ± 0.015	0.172 ± 0.018	0.235 (0.009, 0.029)	0.0005	3.758
$E_{\mathrm{loc}}$	0.283 ± 0.012	0.253 ± 0.012	0.593 (–0.037, 0.022)	<0.0001	8.209
γ	0.769 ± 0.120	0.680 ± 0.115	0.187 (0.185,0.159)	0.0150	2.585
σ	0.628 ± 0.082	0.526 ± 0.031	0.403 (0.467,0.156)	0.0009	3.856
λ	0.518 ± 0.012	0.545 ± 0.032	0.192 (–0.044, 0.008)	0.0060	2.923

Metrics are expressed as the mean ± standard deviation. Abbreviations: SMD, standardized mean difference; CI, confidence interval; Lp, shortest path length; $E_{\mathrm{glob}}$, global efficiency; $E_{\mathrm{loc}}$, local efficiency.

**Fig. 8:**
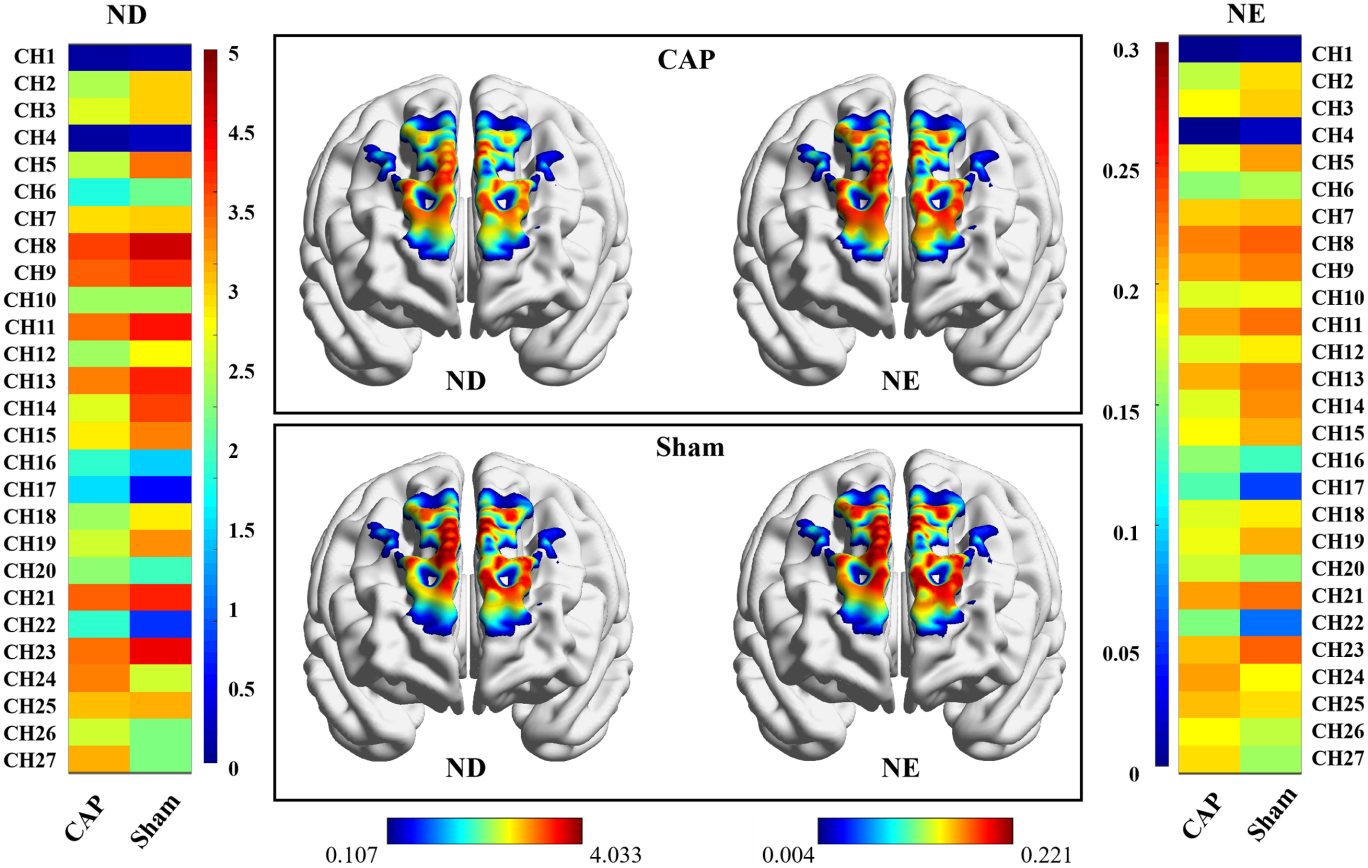
Visual map of regional nodal properties of PFC in CAP and Sham. The color in each topological plot represents the level of ND and NE in the network. ND and NE were calculated by taking the averages of all subjects in each group. The ND and NE of each channel are shown in the heatmap on both sides of the topological scalp plot, with channel labels indicating their locations.

The regional nodal functions were visualized in Fig. 8 and Table 4. The patterns of ND and NE were clearly different between CAP and Sham. Compared with Sham, CAP showed decreased NE in mpfc (p<0.05). Meanwhile, CAP showed decreased NE and ND in ldlpfc (p<0.01). However, the node attributes among all other ROIs did not show significant differences.

**Table 4:** Alterations in the regional nodal functions were identified in three ROIs of CAP.

ROIs	ND/NE	Sham	CAP	p	t
mpfc	NE	0.175 ± 0.067	0.156 ± 0.081	0.0257	2.305
ldlpfc	NE	0.156 ± 0.081	0.169 ± 0.089	0.0033	2.194
ldlpfc	ND	2.547 ± 1.648	2.965 ± 1.888	0.0063	2.862

Data are presented as the mean ± standard deviation. Abbreviations: mpfc, medial prefrontal cortex; ldlpfc, left dorsolateral prefrontal cortex; NE, nodal efficiency; ND, nodal degree

## 4 Discussion

This study used brain FC to examine the strength of pairwise interactions between channels and ROIs in the PFC. As expected, healthy individuals have stronger FC. Interestingly, our study showed that there was a greatly significant difference in connectivity associated with the ldlpfc. Compared with Sham, CAP had impaired functions in both short- and long-range connectivity. After studying all the pairwise interactions between channels and ROIs, we found that the FC of ldlpfc performed well in distinguishing between Sham and CAP. Using graph theoretical analysis, we further delved into the widespread network-level pathophysiological characteristics of CAP at the level of topological indicators. Despite the small number of nodes in this study, we ensured the robustness of the analysis results using methods such as sparsity thresholding and AUC calculation. It has been shown that even with a small number of nodes, fNIRS studies can effectively reveal the topological properties of functional brain networks.^40^^,^^41^ We found that CAP showed impaired small-world properties at a global level compared with that of Sham. At the regional level, two brain regions were identified as showing significant differences among groups in NE.

### 4.1 Widely Disrupted Functional Connectivity

Local application of capsaicin or intradermal injection of capsaicin has been described as a model of neuropathic pain, which produces spontaneous burning pain and local nociceptive hypersensitivity.^23^ With the continuous development of neuroimaging techniques, network-based conceptual frameworks have been used to study the pathogenesis and recovery from pain. The dynamic interactions between multiple cortical and subcortical networks differ to varying degrees among those experiencing different pain types.^54^^–^^56^ Our study investigated channel-based and ROI-based FC. Consistent with previous studies, we found impaired FC in the PFC of the brain during pain.^57^^–^^59^

In addition, we looked at the short- and long-distance connectivity in CAP. Short- and long-distance connectivity could have a different role in establishing brain function, which indicates that damage to either one could have different effects.^60^^,^^61^ Capsaicin-induced pain is usually a localized, acute sensory experience that primarily involves the source of the painful stimulus. Due to the characterization and perception of pain signals, the nervous system may rely more on short-distance connections for rapid local processing. This makes the reorganization of short-distance connections appear more significant, whereas functional changes in long-distance connections may be relatively minor because of tighter signaling among local nodes.

### 4.2 Global Topological Dysfunction of Networks in CAP

The reduction of FC across the brain during pain is accompanied by alterations in the brain networks’ inherent topological structure. Brain regions integrate and distribute information through powerful interconnected networks. A network is considered to have small-world properties if its σ value is greater than 1, which is believed to represent an optimal balance between network segregation and integration.^62^ In our study, the functional brain networks of CAP exhibited efficient small-world topology (σ>1) in the sparsity range (0.05 to 0.50), which was consistent with previous neuroimaging studies of different pain diseases.^55^^,^^63^^,^^64^ Moreover, we found that CAP had decreased the γ and σ values but increased the λ values compared with that of Sham. Anomalies in the small-world properties of CAP suggest that local efficiency, fault tolerance, and the brain’s information-carrying capacity are disrupted. These abnormalities may be related to the loss of remote communication among brain parts.

The Eg is an important metric used to rate the efficiency of information transfer in the network, which directly reflects the effectiveness of the brain in handling information exchange and resource allocation.^65^ The Lp is defined as the number of edges on the shortest path moving from one node to another, reflecting all of the possible channels of information transmission between two brain regions.^66^ Together, Eg and Lp provide complementary insights into the integration of network functions: Eg captures the global efficiency of information transfer across the entire network, whereas Lp quantifies the ease of communication among specific brain regions. Functional integration is achieved through complex connections and signaling among neurons, which allows different parts of the brain to work together to engage and process information for efficient cognitive and behavioral responses. Abnormal reductions in functional integration (lower Eg and higher Lp) are indicative of decreased efficiency of information transfer among different regions of the human brain.

In our study, the $E_{\mathrm{loc}}$ was significantly decreased in CAP compared with Sham. The $E_{\mathrm{loc}}$ measures the information transfer efficiency of local subgraphs (usually those formed by a node’s direct neighbors) in a network. Specifically, $E_{\mathrm{loc}}$ is concerned with the closeness of connections and information transfer capability between a node and its direct neighbors. Lower $E_{\mathrm{loc}}$ means decreased local information processing, which indicates a change in the optimal topological organization of the functional networks. Several studies have also exhibited disrupted topological organization of functional networks in individuals with pain compared with healthy subjects.^67^^–^^69^

Our results indicate that parallel information transfer in brain functional networks is impaired, and the small-world attributes (γ, σ, and λ), Lp, $E_{\mathrm{loc}}$, and Eg may have the potential to be used as biomarkers to monitor the course of the disease as well as to assess the severity of the conditions in painful conditions.

### 4.3 Regional Topological Dysfunction of Networks in CAP

At the regional level, we found decreased NE mainly in the mpfc and ldlpfc and decreased ND in the ldlpfc. This indicates that under the influence of pain, brain networks became more fragmented, with nodes acting as fewer shortest path hubs of other nodes, and therefore, information exchange within the network tended to be more indirect and less efficient.^55^ For pain individuals, this can mean that their ability to cope with pain, regulate their emotions, or perform cognitive tasks is compromised. In line with these studies, we found that these regions had lower NE in chronic pain patients than in healthy subjects.^70^^–^^72^ Even though the NE and ND were reduced in the pain individuals, their brain networks continued to exhibit small-world properties, suggesting that the kind of efficient network structure with an optimal balance between network segregation and integration observed in the normal human brain is maintained in the pain state.^73^ The small-world properties were preserved in the functional brain networks of neuropathic pain patients, whether due to brachial plexus injury^74^ or postherpetic neuralgia.^75^ This suggests that, in contrast to other neurological or psychiatric conditions like Alzheimer’s disease or schizophrenia, the remodeling process of the brain after chronic neuropathic pain is relatively subtle and does not significantly disrupt the small-world properties and structural integrity of brain networks.^73^

### 4.4 Limitations

The present study has several limitations that need to be improved in future studies. First, the HbR signal also contains information about the cortical pain response, but this was not quantitatively analyzed in the current study. Estimating the similarity between HbO and HbR networks using methods such as DSI^76^ may help to further elucidate the cortical mechanisms of pain response. Second, pain triggers changes in physiological information, such as heart rate and blood pressure, which affect systemic responses inside and outside the brain. Therefore, in future studies, we will add short separation channels to better eliminate the effects of physiological signals.^77^^–^^79^ In addition, synchronizing the acquisition of physiological information will help reduce spurious connections that may result from the interaction of physiological information with cortical blood flow. Third, this study only monitored fNIRS in the forebrain cortex and did not cover the entire cerebral cortex. Future studies could adjust the number of device channels to provide a more comprehensive view of the dynamic changes in the brain during the experience of pain.

## 5 Conclusion

In the present study, topical application of capsaicin produced burning pain with a corresponding increase in pain scores. From a network topological perspective, CAP and Sham have different topological architecture models and nodal functions in PFC. Using graph theoretical analysis, we explored brain connectivity in CAP in the resting state. It was found that the functional brain networks of CAP suffered from impaired properties at both the holistic and nodal levels compared with healthy subjects. Notably, CAP showed a decrease in short- and long-distance connectivity. Even brief episodes of acute pain can significantly reshape the brain’s network architecture and FC, revealing a multifaceted phenomenon that transcends a mere fleeting sensory event. The alterations in brain network topology and connectivity caused by pain reveal the potential for implementing targeted therapeutic strategies and suggest a reorganization of neural pathways that may lead to the development of persistent pain. This work not only advances our understanding of pain mechanisms but also opens new avenues for targeted interventions and therapies.

## Supplementary Material

10.1117/1.NPh.12.2.025010.s01

## Data Availability

The code used for fNIRS data analyses is publicly available on GitHub at the following URL: https://github.com/Yijing719/Pain-re-fNIRS.git. All data supporting the findings of this paper are publicly available in an Open Science Framework repository titled “Cortical functional connectivity and topology based on complex network graph theory analysis during acute pain stimuli,” at DOI https://osf.io/tc4g5/files/osfstorage.
