# Supplementary material for: Cortical functional connectivity and topology based on complex network graph theory analysis during acute pain stimuli
Source: Neurophotonics. 2025 May 14;12(2):025010. doi: 10.1117/1.NPh.12.2.025010 (PMC12077576; doi:10.1117/1.NPh.12.2.025010)
Supplement: Supplementary file 1 [file NPh_012_025010_SD001.pdf]

Functional connectivity analyses of the HbR signals were performed which revealed no significant differences between the two groups (CAP vs. Sham: 0.37 ± 0.03 vs. 0.41 ± 0.01, $p>0.05$, see Supplementary Material 1).

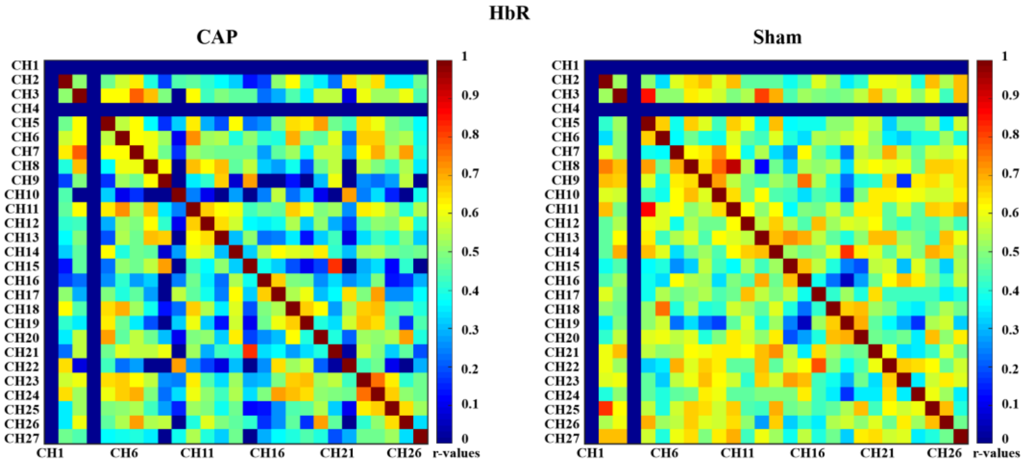

Figure S1 Group-average correlation matrix of HbR signals for CAP and Sham. The colour bar indicates the r-values of functional connectivity strength, with higher values indicating greater connection strength.
